# A Novel Bromophenol Derivative BOS-102 Induces Cell Cycle Arrest and Apoptosis in Human A549 Lung Cancer Cells via ROS-Mediated PI3K/Akt and the MAPK Signaling Pathway

**DOI:** 10.3390/md16020043

**Published:** 2018-01-25

**Authors:** Chuan-Long Guo, Li-Jun Wang, Yue Zhao, Hua Liu, Xiang-Qian Li, Bo Jiang, Jiao Luo, Shu-Ju Guo, Ning Wu, Da-Yong Shi

**Affiliations:** 1 Key Laboratory of Experimental Marine Biology, Institute of Oceanology, Chinese Academy of Sciences, Qingdao 266071, China; gcl_cpu@126.com (C.-L.G.); wanglijun@qdio.ac.cn (L.-J.W.); zhaoyue19931104@163.com (Y.Z.); baihualin55100@163.com (H.L.); lnu101@163.com (X.-Q.L.); jiangbo@qdio.ac.cn (B.J.); luojiao2012@163.com (J.L.); guoshuju@qdio.ac.cn (S.-J.G); wuning@qdio.ac.cn (N.W.); 2 Laboratory for Marine Drugs and Bioproducts, Qingdao National Laboratory for Marine Science and Technology, Qingdao 266071, China; 3 University of Chinese Academy of Sciences, Beijing 10049, China

**Keywords:** bromophenol, molecular mechanisms, apoptosis, cell cycle, PI3K/Akt, p38/ERK, ROS, human lung cancer

## Abstract

Bromophenol is a type of natural marine product. It has excellent biological activities, especially anticancer activities. In our study of searching for potent anticancer drugs, a novel bromophenol derivative containing indolin-2-one moiety, 3-(4-(3-([1,4′-bipiperidin]-1′-yl)propoxy)-3-bromo-5-methoxybenzylidene)-N-(4-bromophenyl)-2-oxoindoline-5-sulfonamide (**BOS**-**102**) was synthesized, which showed excellent anticancer activities on human lung cancer cell lines. A study of the mechanisms indicated that **BOS**-**102** could significantly block cell proliferation in human A549 lung cancer cells and effectively induce G0/G1 cell cycle arrest via targeting cyclin D1 and cyclin-dependent kinase 4 (CDK4). **BOS**-**102** could also induce apoptosis, including activating caspase-3 and poly (ADP-ribose) polymerase (PARP), increasing the Bax/Bcl-2 ratio, enhancing reactive oxygen species (ROS) generation, decreasing mitochondrial membrane potential (MMP, Δ*Ψ*_m_), and leading cytochrome c release from mitochondria. Further research revealed that **BOS**-**102** deactivated the PI3K/Akt pathway and activated the mitogen-activated protein kinase (MAPK) signaling pathway resulting in apoptosis and cell cycle arrest, which indicated that **BOS**-**102** has the potential to develop into an anticancer drug.

## 1. Introduction

In China, cancer morbidity and mortality are increasing year by year. It has become the leading cause of death and seriously affected the quality of life of people with cancer [1]. Lung cancer is the cancer with the highest mortality rate in the world [1,2,3]. Among lung cancers, approximately 85% of cases are non-small cell lung cancer (NSCLC). Thus, it is highly desirable to develop safe and effective drugs to treat NSCLC and to improve the quality of life of patients with lung cancer. 

Apoptosis can occur by one of two representative pathways: the mitochondrial pathway and the death receptor-mediated pathway [4]. In the mitochondrial pathway, ROS plays an important role [5]. Generated ROS directly activates the mitochondrial permeability transition and results in mitochondrial membrane potential (MMP, Δ*Ψ*_m_) loss, causing the release of cytochrome c. Then the released cytochrome c activates caspases, and the activated caspases eventually lead to apoptosis [6,7,8]. In addition, ROS could also induce other various signaling pathways, such as the PI3K/Akt signaling pathway, which plays an important role in cell proliferation and survival [9]. When cells are subject to external stimuli, such as anticancer drug stimulation, the PI3K/Akt signaling pathway may be down-regulated, and finally induce cell apoptosis. The PI3K/Akt pathway also plays an important role in cell cycle regulation; the mechanism of this pathway is the regulation of CDK4 and cyclin D1, which could block cell cycle in G1 phase [10]. 

ROS is a second messenger in cells, and plays an important role in cell proliferation or apoptosis [11]. It has been demonstrated that ROS generation in cells could activate mitogen-activated protein kinase (MAPK) signaling pathways including p38 MAPK and ERK1/2 [12]. MAPK pathways are key mediators of eukaryotic transcription and they control gene expression via phosphorylation [13]. 

Bromophenols, an important kind of natural marine product, extracted from a variety of marine organisms, exhibit excellent biological activities, such as antitumor and antibacterial activities [14,15,16]. However, the natural bromophenol in the marine organisms is in low content, and its separation is difficult. Additionally, there are more or less deficiencies in its efficacy, stability, biological toxicity, and bioavailability. These are extremely limited for its further research and development [15].

**Figure 1:**
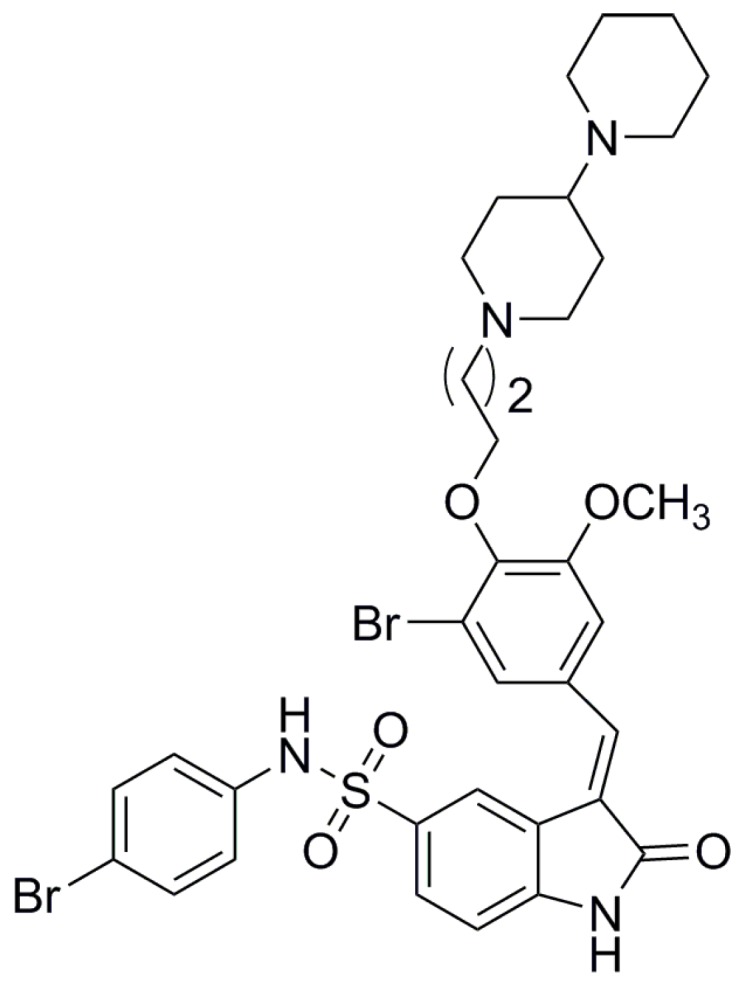
The structure of **BOS**-**102**.

Synthesis of new bromophenol compounds, or modifications to the structure of natural bromophenols, can effectively improve the biological activity of bromophenol compounds. Based on this foundation, a series of bromophenol compounds with potent anticancer activities were designed and synthesized in our previous works [14]. These compounds showed anticancer activity on human cancer cell lines, such as the human lung cancer cell line, human hepatoma cell lines, the human cervical cancer cell line, and the human colon cancer cell line. Among of them, a novel bromophenol derivative containing indolin-2-one moiety, 3-(4-(3-([1,4′-bipiperidin]-1′-yl)propoxy)-3-bromo-5-methoxybenzylidene)-*N*-(4-bromophenyl)-2-oxoindoline-5-sulfonamide (**BOS**-**102, Figure 1**) showed excellent anticancer activities on human lung cancer cell lines [17]. In order to study whether compound **BOS**-**102** has the potential to develop into an anticancer drug, more experiments regarding the molecular mechanisms, including cell apoptosis analysis, cell cycle analysis, ROS generation analysis, mitochondrial membrane potential analysis, and potential signaling pathways (PI3K/Akt and MAPK) analysis in A549 lung cancer cells were explored in this study. 

## 2. Results

### 2.1. **BOS**-**102** Inhibits Cell Proliferation 

**Figure 2:**
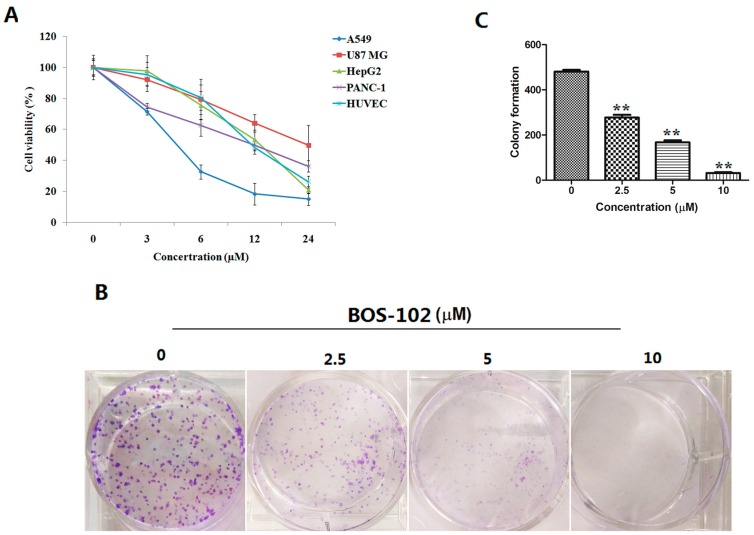
**BOS**-**102** inhibits the viability, migration, and colony formation in human cancer cell lines. (**A**) The inhibitory effect of **BOS**-**102** on the cell proliferation of A549, HepG2, U87 MG, and PANC-1 cells. Cells were treated with various concentrations of **BOS**-**102** (0, 3, 6, 12, 24 µM) for 48 h. After incubation, cell viability was evaluated by MTT assay; (**B**,**C**) A549 cells were treated with **BOS**-**102** (0, 2.5, 5, 10 µM) for 10 day and colony formation was determined by staining with crystal violet. The data represent mean values (±SD) obtained from three separate experiments. ** *p* < 0.01 vs. control group.

We examined the effect of **BOS**-**102** on cell viability using MTT assay. The results suggested that **BOS**-**102** could induce cytotoxicity of several tumor cell lines, including human lung cancer cell line A549, human hepatoma cell line HepG2, human primary glioblastoma cell line U87 MG, and human pancreatic cell line PANC-1. **BOS**-**102** could also induce cytotoxicity on normal cells, such as human umbilical vein endothelial cells (HUVECs). As shown in Figure 2A, **BOS**-**102** could inhibit cell proliferation of cancer cell lines, including A549 (IC_50_ = 4.29 ± 0.79 µM), HepG2 (IC_50_ = 13.87 ± 1.40 µM), U87 MG (IC_50_ = 23.98 ± 8.80 µM), PANC-1 (IC_50_ = 12.48 ± 1.66 µM), and HUVECs (15.43 ± 1.07 µM). In our study, A549 cells were much more sensitive. Thus, A549 cells were used in the subsequent experiments. 

### 2.2. **BOS**-**102** Inhibits Colony Formation

A colony formation assay was performed to study the effect of **BOS**-**102** on cell colony formation. A549 cells were seeded in six-well plates at a density of 500 cells per well. After 24 h, cells were treated with **BOS**-**102** (0, 2.5, 5, 10 μM), and incubated for 10 days. In our study, the results showed that **BOS**-**102** can significantly inhibit the colony formation of A549 cells (Figure 2B,C).

### 2.3. **BOS**-**102** Induces A549 Apoptosis 

**Figure 3:**
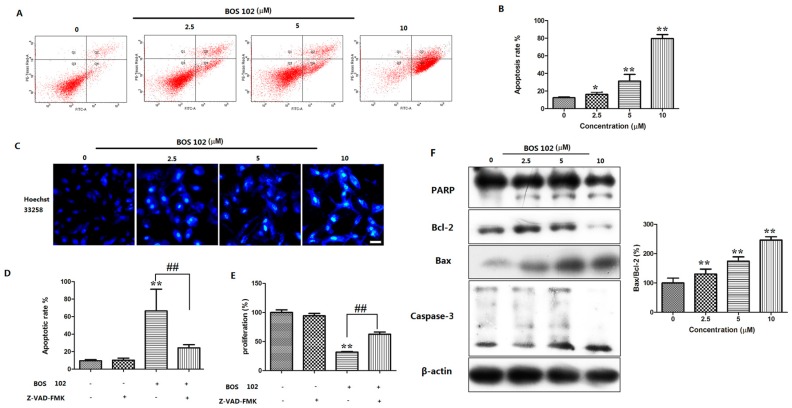
**BOS**-**102** induces intrinsic apoptosis in A549 cells. (**A**,**B**) FACS analysis via Annexin V/PI staining was used to identify apoptosis induced by **BOS**-**102**. A549 cells were treated with various concentrations of **BOS**-**102** (0, 2.5, 5, 10 µM) for 48 h; (**C**) A549 cells were treated with **BOS**-**102** (0, 2.5, 5, 10 µM) for 48 h. Hoechst 33258 staining was used to detected the apoptosis and photographed using fluorescence microscopy (Bar = 50 µm); (**D**) A549 cells were treated with 5 µM **BOS**-**102** alone or in combination with Z-VAD-FMK (10 µM) for 48 h. The percentages of apoptotic cells were determined by flow cytometr (FACS) analysis via Annexin V/PI staining; (**E**) A549 cells were treated with 5 µM **BOS**-**102** alone or in combination with Z-VAD-FMK (10 µM) for 48 h, cell viability was evaluated by MTT assay; and (**F**) Western blot analysis of apoptosis-related proteins, including PARP, Bcl-2, Bax, and Caspase-3. β-actin was used to normalize the protein content. The data represent mean values (±SD) obtained from three separate experiments. * *p* < 0.05, ** *p* < 0.01 vs. control group, ^##^ *p* < 0.01 vs. 102(+)/Z-VAD-FMK(−) group.

To evaluate effect of **BOS**-**102** on the induction of apoptosis, A549 cells were treated with **BOS**-**102** (0, 2.5, 5, 10 μM) for 48 h. After stained with Annexin V/PI, cells were analyzed by flow cytometry. As shown in Figure 3A,B, **BOS**-**102** induced apoptosis in A549 cells in a concentration-dependent manner. Compared with treatment of **BOS**-**102** at 2.5 μM, the percentage of apoptotic cells was increased from 16.2 ± 2.5% to 79.2 ± 4.5% after treatment with **BOS**-**102** at 10 μM (Figure 3A,B). Moreover, Z-VAD-FMK (the pan-caspase inhibitor) was used in our study. The results showed that Z-VAD-FMK could inhibit **BOS**-**102**-induced apoptosis (Figure 3D) and **BOS**-**102**-induced cytotoxicity in A549 cells (Figure 3E). 

Apoptosis often causes cell morphological changes, such as nuclear apoptotic bodies [18]. It is interesting to investigate the effect of **BOS**-**102** apoptosis induction by Hoechst 33258 staining in the A549 cell line. A549 cells were treated with **BOS**-**102** (0, 2.5, 5, 10 μM) for 48 h. As shown in Figure 3C, after staining with Hoechst 33258, cell nuclear condensation, chromosome condensation, and apoptotic bodies were observed in **BOS**-**102**-treated cells.

### 2.4. Effect of **BOS**-**102** on the Expression of Apoptosis-Related Proteins

When apoptosis occurred, the expression of apoptosis related proteins, such as Bax, Bcl-2, caspase-3, and PARP may change. Western blot was used to detect the expression of these proteins. After treatment with **BOS**-**102** for 48 h, the expression of Bax was increased while the Bcl-2 was decreased (Figure 3F). Furthermore, caspase-3 and PARP were also activated after **BOS**-**102** treatment (Figure 3F). Our results indicated that **BOS**-**102** induced apoptosis on A549 cells probably through the mitochondrial-mediated apoptotic pathway.

### 2.5. **BOS**-**102** Induces G0/G1 Cell Cycle Arrest and Down-Regulates Cyclin D1 and CDK4 in A549 Cells

**Figure 4:**
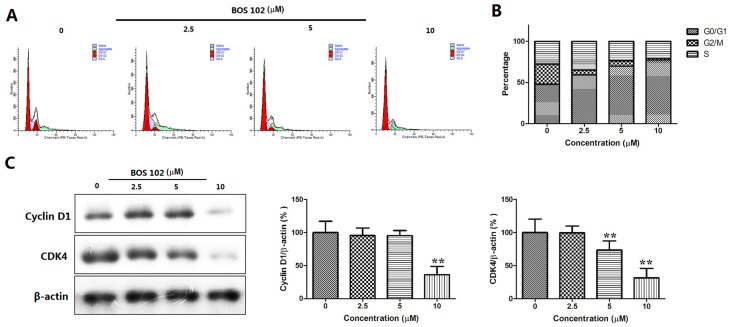
**BOS**-**102** induces G0/G1 cell cycle arrest. (**A**,**B**) Cell cycle distribution was monitored by FACS. A549 cells were treated with various concentrations of **BOS**-**102** (0, 2.5, 5, 10 µM) for 48 h. Cells were harvested and fixed in 70% ethanol overnight, then cells were stained with PI and analysis by FACS; and (**C**) Western blot analysis of cell cycle-related proteins, including Cyclin D1 and CDK4. β-actin was used to normalize protein content. The data represent mean values (±SD) obtained from three separate experiments. ** *p* < 0.01 vs. control group.

To investigate the effects of **BOS**-**102** on cell cycle distribution, A549 cells were treated with **BOS**-**102** (0, 2.5, 5, 10 μM) for 48 h and analyzed by flow cytometry. The results showed that the G0/G1 phase was increased in a dose-dependent manner after **BOS**-**102** treatment. (Figure 4A,B). Treatment with **BOS-12** for 48h caused a remarkable dose-dependent accumulation of cells in G0/G1 phase; from 46.06% (0 μM) to 74.37% (10 μM), these findings denoted that **BOS**-**102** could induce G0/G1 cell cycle arrest.

To explore the mechanism for **BOS**-**102**-induced cell cycle arrest, Western blot analysis was used to examine the effect of **BOS**-**102** treatment on the expression levels of cell cycle-related proteins, such as cyclin D1 and CDK4. In our study, **BOS**-**102** treatment caused a significant decrease in the protein levels of cyclin D1 and CDK4 (Figure 4C). 

### 2.6. **BOS**-**102** Induces Mitochondrial Dysfunctionin in A549 Cells

**Figure 5:**
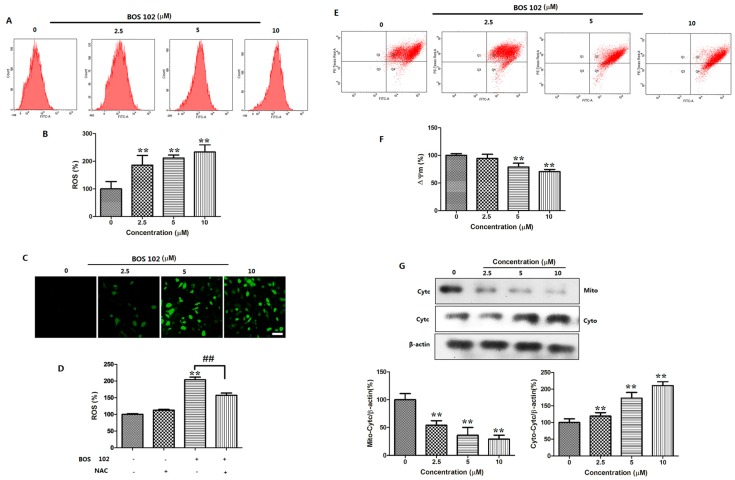
**BOS**-**102** induces ROS generation and loss of MMP in A549 cells. (**A**–**C**) A549 cells were treated with various concentrations of **BOS**-**102** (0, 2.5, 5, 10 µM) for 48 h, the medium was discarded, and cells were incubated at 37 °C in the dark for 20 min with culture medium containing DCFDA. Cells were harvested and analysis using FACS and fluorescence microscopy (Bar = 50 µm); (**D**) A549 cells were treated with 5 µM **BOS**-**102** alone or in combination with NAC (5 mM) for 48 h. ROS generation was detected by FACS after staining with DCFDA; (**E**,**F**) A549 cells were treated with various concentrations of **BOS**-**102** (0, 2.5, 5, 10 µM) for 48 h. The cells were collected and incubated with JC-1 for 20 min at 37 °C, cells were then washed twice with PBS and the values of MMP were analyzed by FACS; (**G**) Immunoblotting of mito-cytochrome c and cyto-cytochrome c. The data represent mean values (±SD) obtained from three separate experiments. ** *p* < 0.01 vs. control group. ^##^ *p* < 0.01 vs. 102(+)/NAC(−) group.

The maintenance of MMP (∆*Ψ*_m_) is significant for mitochondrial integrity and bioenergetic function [19]. The decline of MMP is a marker of apoptosis. We used JC-1 to detect the decline of MMP of A549 cells after **BOS**-**102** treatment. In this study, the results indicated that **BOS**-**102** significantly reduced the MMP in A549 cells compared with the control group (Figure 5E,F). Additionally, we detected the expression of cytochrome c in mitochondria and cytoplasm using Western blot analysis. The results indicated that **BOS**-**102** decreased cytochrome c in mitochondria, while increased it in cytoplasm (Figure 5G). The results indicated that **BOS**-**102** induced apoptosis in A549 cells through the mitochondrial apoptotic pathway.

### 2.7. **BOS**-**102** Induces ROS Generation in A549 Cells

ROS generation is considered as one of the key mediators of apoptotic signaling. We used DCFH-DA to detect ROS in A549 cells. In our study, a rapid production of ROS was occurred after the treatment of **BOS**-**102** (0, 2.5, 5, 10 μM) for 48 h. The result showed that DCFH-DA fluorescence intensity was increased in a dose-dependent manner. Compared with control group, the content of ROS was increased to 185.84 ± 24.91%, 211.50 ± 7.69%, and 233.36 ± 18.52% (Figure 5A–C). Next, A549 cells were treated with 5 μM **BOS**-**102** combined with/without 5 mM *N*-acetyl cysteine (NAC) for 48 h, respectively. Interestingly, we observed that the ROS inhibitor NAC blocked ROS generation in A549 cells (Figure 5D). We also observed that NAC blocked **BOS**-**102**-induced apoptosis in A549 cells (data not shown). These results indicated that ROS generation is important in **BOS**-**102**-induced apoptosis.

### 2.8. **BOS**-**102** Suppresses the PI3K/Akt Signaling Pathway

**Figure 6:**
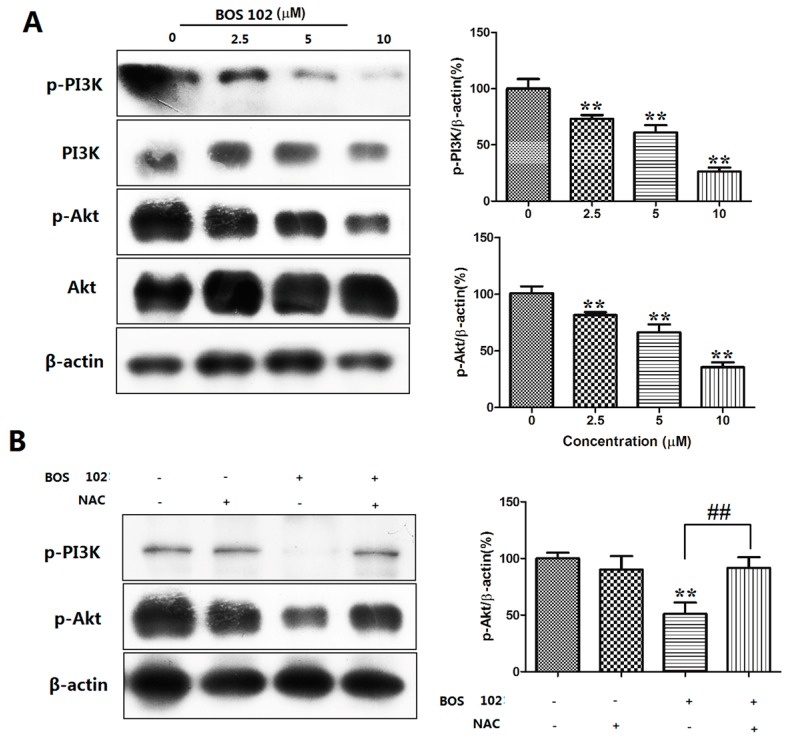
Effects of **BOS**-**102** on PI3K/Akt pathway. (**A**) A549 cells were treated with various concentrations of **BOS**-**102** (0, 2.5, 5, 10 µM) for 48 h and then the expressions and phosphor of PI3K and Akt were assessed by western blot analysis; and (**B**) the effect of antioxidant NAC on **BOS**-**102**-induced Akt activation. All data were representative of three independent experiments. ** *p* < 0.01 vs. control group. ^##^ *p* < 0.01 vs. 102(+)/NAC(−) group.

When apoptosis occurs, the PI3K/Akt signaling pathway plays an important role, which is also an important modulator in ROS-related cell apoptosis. In our study, we used Western blot analysis to detect the expression levels of PI3K/Akt after treatment of **BOS**-**102**. As shown in Figure 6A, the phosphorylation of Akt and PI3K were decreased in a dose-dependent manner after **BOS**-**102** treatment. Furthermore, pretreatment of antioxidant NAC, a ROS inhibitor, could efficiently reverse the **BOS**-**102**-induced Akt phosphorylation (Figure 6B). These results indicated that **BOS**-**102** induced cell apoptosis by inhibiting the PI3K/Akt signaling pathway.

### 2.9. **BOS**-**102** Activates the P38/ERK Signaling Pathway 

**Figure 7:**
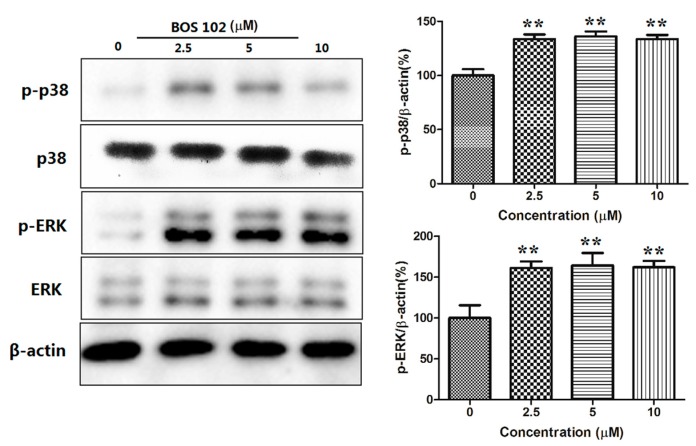
Effects of **BOS**-**102** on the MAPK pathway. A549 cells were treated with various concentrations of **BOS**-**102** (0, 2.5, 5, 10 µM) for 48 h and then the expressions and phosphor of p38 and ERK were assessed by Western blot analysis. All data were representative of three independent experiments. ** *p* < 0.01 vs. control group.

It is well known that the MAPK signaling pathway plays a critical role in regulating cell apoptosis. The effect of **BOS**-**102** on this pathway was detected using Western blot. As shown in Figure 7, at various concentrations after application of **BOS**-**102**, the phosphorylation of ERK and p38 were significantly increased. These results indicated that the ERK/p38 signaling pathway was involved in **BOS**-**102**-induced apoptosis.

## 3. Discussion

Non-small cell lung cancer is the most common form of lung cancer globally. Currently, surgery and radiotherapy are available therapeutic approaches for lung cancer patients, but both of them cause severe pain and side-effects [2]. It is highly desirable to develop safe and effective drugs to treat lung cancer and to reduce the pain of patients with lung cancer. In the current study, we reported that **BOS**-**102** could inhibit the proliferation of several cancer cell lines, including human lung cancer cell line A549 (IC_50_: 4.29 ± 0.79 µM), human hepatoma cell line HepG2 (IC_50_: 13.87 ± 1.40 µM), human primary glioblastoma cell line U87 MG (IC_50_: 23.98 ± 8.80 µM) and human pancreatic cell line PANC-1 (IC_50_: 12.48 ± 1.66 µM). In addition, **BOS**-**102** could inhibit the proliferation of normal cells such as human umbilical vein endothelial cells (HUVECs). In the following experiment, we demonstrated that **BOS**-**102** significantly inhibited colony formation in A549 cells (Figure 2B,C), which was largely due to the effect of **BOS**-**102** induced G0/G1 cell cycle arrest and apoptosis. Furthermore, we investigated the molecular mechanisms of anti-cancer effect of **BOS**-**102**, which was likely mediated through various signaling pathways. 

As we all know, there are two main methods for cell growth inhibition: cell cycle arrest and apoptosis. In mammalian cells, cell-cycle progression is controlled by a series of cyclin-dependent kinase (CDK)–cyclin complexes. CDK family members, such as CDK2, CDK4, and CDK6, play a key role in the cell-cycle control of tumor cells [20]. Our results showed that the proportion in the G1 phase was significantly increased from 46.06% to 74.37% after treatment of **BOS**-**102** for 48 h (Figure 4A,B), denoting that **BOS**-**102** induced G0/G1 arrest in A549 cells. Furthermore, the mechanisms of **BOS**-**102**-induced cell cycle arrest were investigated using Western blot analysis. Our results showed that the expression of cyclin D1 and CDK4 was decreased after treatment of **BOS**-**102** in A549 cells (Figure 4C). As we all know, activation of cyclin D1 is important when cells were transiting through G1 into the S phase, then the activated cyclin D1 binds to CDK4 and, finally, induces cells from G1 to the S phase [21,22]. In our study, the expression of cyclin D1 and CDK4 were decreased after treatment of **BOS-102,** indicating that **BOS**-**102** could exert its anti-proliferative effect via inducing G0/G1 cell cycle arrest, and the molecular mechanisms may be through the modulation of cyclin D1 and CDK4.

Apoptosis is a normal physiological phenomenon that can be observed in various tissues and cells. When apoptosis happens, various morphological changes will appear, such as cell surface changes, nuclear pyknosis, DNA fragmentation, and chromosome condensation [23]. Morphological changes in A549 cells were analyzed by Hoechst 33258 staining (Figure 3C). The phosphatidylserine externalization (cell surface changes) in A549 cells was detected by flow cytometry after staining with Annexin V-FITC/PI, and our results showed that **BOS**-**102** induced A549 apoptosis in a concentration-dependent manner (Figure 3A,B). In order to detect whether caspase was involved in **BOS**-**102**-induced apoptosis, we used the pan-caspase inhibitor, Z-VAD-FMK, which can inhibit caspase processing and apoptosis induction in tumor cells in vitro. In our study, Z-VAD-FMK inhibited **BOS**-**102**-induced A549 apoptosis and cell hypoproliferation (Figure 3D). These experiments indicated that **BOS**-**102** could induce apoptosis in A549 cells via a caspase-dependent pathway. 

It is well known that apoptosis can be activated by various stressors, among which ROS is the major cause of toxicity. It has been reported that ROS acts as a second messenger in the signal process [7,24]. ROS can induce apoptosis via a variety of mechanisms. For instance, increased ROS can active the intrinsic pathway by stimulating the depolarization of MMP [25]. The depolarization of MMP can activate the intrinsic apoptosis pathway and be tightly regulated bythe Bcl-2 family, such as Bcl-2 (anti-apoptotic protein) and Bax (pro-apoptotic protein) [26]. In the present study, an increase of ROS generation was observed in the **BOS**-**102**-treated A549 cells (Figure 5A–C). Furthermore, the ROS generation ability of **BOS**-**102** could be inhibited by NAC, a specific inhibitor of ROS (Figure 5D). Our results also clearly indicated that MMP was collapsed after treatment of **BOS**-**102** (Figure 5E,F). As we all know, the loss of MMP leads to cytochrome c release, and the released cytochrome c can activate caspase-3 and PARP. In this study, after **BOS**-**102** treatment, the expression of Bax was increased while Bcl-2 was decreased (Figure 3E). Additionally, cytochrome c was released to cytoplasm (Figure 5G), resulting in caspase and PARP activation (Figure 3F) and cell apoptosis (Figure 3A,B). In general, our data demonstrated that **BOS**-**102** could induce A549 cells apoptosis via the ROS-mediated mitochondria pathway.

The PI3K/Akt pathway plays an important role in regulating cell survival and death [27]. Therefore, the suppression of the PI3K/Akt signaling pathway may be an effective approach to the treatment of human lung cancer. Phosphorylated Akt can promote the apoptosis-related protein Bcl-2 and cell cycle-related protein cyclin D. Our study found that **BOS**-**102** treatment could induce decreased phosphorylation of PI3K and Akt (Figure 6A). In our previous study, our results suggested that ROS generation played an important role in **BOS**-**102**-induced apoptosis, whether the PI3K/Akt pathway was also connected with ROS generation. In the following study, pretreatment of NAC could efficiently reverse the **BOS**-**102**-induced PI3K and Akt phosphorylation (Figure 6B), suggesting the PI3K/Akt pathway might also be implicated in **BOS**-**102**-induced apoptosis, and this process was via excessive generation of ROS.

Mitogen-activated protein kinase (MAPK) signaling pathways also play an important role in the development of cancer. An increase in intracellular ROS can activate MAPK signaling pathways, including p38 MAPK and ERK1/2 [12]. It has been demonstrated that p38 and ERK play a vital role in cell survival and apoptosis [13]. On the one hand, some literatures reported that ERK activation was beneficial to cell proliferation and survival [28]. On the other hand, there is also some literature reported that ERK activation could induce cell apoptosis [4,13,29]. In our study, we found that the phosphorylation of p38 and ERK were increased after treatment of **BOS**-**102** (Figure 7), indicating that the MAPK signaling pathway was also related to **BOS**-**102**-induced apoptosis in A549 cells.

## 4. Materials and Methods 

### 4.1. Reagents

**BOS**-**102** was synthesized by our lab according to our previous publish [17], with a purity of 98%. The purity of compound **BOS**-**102** was measured by HPLC (Shimadzu, Kyoto, Japan), carried out on a Shimadzu LC-20A system (Shimadzu, Kyoto, Japan) equipped with a Shimadzu InertSustain C-18 reverse phase column (4.6 mm × 250 mm × 5 μm, Shimadzu, Kyoto, Japan) and SPD-20A detector (Shimadzu, Kyoto, Japan). In this study, **BOS**-**102** was dissolved in dimethyl sulfoxide (DMSO) and shored in −20 °C for less than one month before use. The vehicle (DMSO) was used as a control group, and the concentration of DMSO used in the experiments was less than 0.1%. The Dulbecco’s modified Eagle’s medium (DMEM) and Eagle’s Minimum Essential Medium (MEM) were obtained from Hyclone (Logan, UT, USA). Fetal bovine serum (FBS) was obtained from ExCell Bio (Shanghai, China). Medium 200 and Low Serum Growth Supplement (LSGS) were obtained from Thermo Fisher Scientific (Waltham, MA, USA). The ROS assay kit, JC-1 assay kit, apoptosis assay kit, and Hoechst 33258 staining kit and *N*-acetyl cysteine (NAC), the pan-caspase inhibitor (Z-VAD-FMK) were obtained from Beyotime (Nanjing, China). Antibodies against cyclin D1, CDK4, Caspase-3, Bcl-2, Bax, PARP, phosphorylation-Akt, Akt, phosphorylation-p38, p38, phosphorylation-ERK1/2, ERK1/2, cytochrome c, and β-actin were purchased from Cell Signaling Technology (Beverly, MA, USA). Phosphorylation-PI3K and PI3K were purchased from Abcam (Cambridge, UK). The anti-mouse IgG and anti-rabbit secondary antibodies raised from goat were obtained from Abcam (Cambridge, UK).

### 4.2. Cell Culture

Human lung cancer cell line A549 cells and human pancreatic cell line PANC-1 cells were cultured in Dulbecco’s Modified Eagle’s medium (DMEM) supplemented with 10% FBS, 100 U/mL penicillin, and 100 U/mL streptomycin. Human hepatoma cell line HepG2 cells and human primary glioblastoma cell line U87 MG cells were cultured in Eagle’s Minimum Essential Medium (MEM) supplemented with 10% FBS, 100 U/mL penicillin and 100 µg/mL streptomycin. Human umbilical vein endothelial cells (HUVECs) were cultured in Medium 200-supplemented LSGS. Cells were cultured at 37 °C in humidified CO_2_ (5%). 

### 4.3. In Vitro Cytotoxicity Test

The cytotoxicity of **BOS**-**102** was tested with standard 3-(4,5-dimethyl-2-thiazolyl)-2,5-diphenyl tetrazolium bromide (MTT) testing. Briefly, cells (A549, HepG2, U87 MG, PANC-1, and HUVECs) were seeded in 96-well plates (3 × 10^3^ cells/well for A549, U87 MG, and PANC-1 cells, 5 × 10^3^ cells/well for HepG2 and HUVEC cells) and allowed to settle 24 h, then cells were treated with varying concentrations of **BOS**-**102** (0, 3, 6, 12, 24 μM). After 48 h, MTT (5 mg/mL) was added in the plates, and incubated at 37 °C for 4 h. After removing the supernatant, the transformed crystals were dissolved in 150 μL DMSO and measured using a microplate reader (BioTek, Winooski, VT, USA) at 490 nm. Experiments were performed in triplicate on six wells for each measurement. 

### 4.4. Colony Forming Assay

A549 cells were seeded in 6-well plates at a density of 500 cells per well. After 24 h, cell were treated with **BOS**-**102** (0, 2.5, 5, 10 μM). Then cells were incubated for 10 days to allow for colony formation. After staining with crystal violet, colonies containing more than 50 cells were counted and evaluated [30].

### 4.5. Assays for Apoptosis

Cell apoptosis analysis was detected using Annexin V/PI staining assay. Briefly, cells (1 × 10^5^ cells/well) were seeded in a six-well plate and incubated for 24 h. Then, cells were treated with **BOS**-**102** (0, 2.5, 5, 10 μM) for 48 h. Cells were harvested, washed with cold PBS, and stained with Annexin V-FITC (Annexin V-Fluorescein isothiocyanate) and PI (propidine iodide). Then cells were analyzed in three different experiments using flow cytometry (Becton Dickinson, Franklin Lakes, NJ, USA). 

### 4.6. Flow Cytometric Analysis of Cell Cycle

A549 cells were seeded into 6-well plates at a density of 5 × 10^5^ cells per well and treated with **BOS**-**102** (0, 2.5, 5, 10 μM) for 48 h. Cells were harvested, fixed in cold 75% ethanol at −20 °C overnight. Cells were washed with PBS, re-suspended with cold PBS containing 20 µg/mL RNaseA and 50 µg/mL PI. After incubation at room temperature in the dark for 30 min. Cells were analyzed in three different experiments using flow cytometry (Becton Dickinson, Franklin Lakes, NJ, USA).

### 4.7. Morphological Analysis of Apoptosis

Cell morphology of apoptosis cells was evaluated by Hoechst 33258 staining. A549 cells were treated with **BOS**-**102** (0, 2.5, 5, 10 μM) for 48 h. Then cells were washed with PBS and stained with Hoechst 33258. After washing twice with PBS, cells were visualized on a fluorescence microscope (Olympus, Tokyo, Japan).

### 4.8. ROS Determination

ROS production was measured after staining with 2′,7′-dichlorohydrofluorescein (DCFH-DA). Briefly, A549 cells (1 × 10^6^) were seed in six-well plates and treated with **BOS**-**102** (0, 2.5, 5, 10 μM). After 48 h incubation, cells were stained with 10 μM DCFH-DA for 20 min at 37 °C in the dark. Then cells were washed with PBS and harvested. Fluorescence was detected on a flow cytometer (Becton Dickinson, Franklin Lakes, NJ, USA) and a fluorescence microscope (Olympus, Tokyo, Japan).

### 4.9. Analysis of the MMP

MMP was assessed using flow cytometry after staining with JC-1. Briefly, A549 cells (1 × 10^6^) were seed in six-well plates and treated with **BOS**-**102** (0, 2.5, 5, 10 μM) for 48 h. Cells were harvested and incubated with JC-1 at 37 °C for 20 min. After two washes with PBS, cells were analyzed using flow cytometry (Becton Dickinson, Franklin Lakes, NJ, USA).

### 4.10. Western Blot Analysis

A549 cells were treated with various concentrations of **BOS**-**102** (0, 2.5, 5, 10 μM) for 48 h. Cells were lysed in RIPA lysis buffer on ice for 15 min. The protein concentrations of the cell lysates were determined with a BCA protein kit (Beyotime, Nanjing, China). Proteins were mixed with loading buffer containing 5% 2-mercaptoethanol and then heated for 5 min at 95 °C. Protein lysates were subsequently loaded into each lane of a 12% SDS–PAGE and transferred onto PVDF membranes. Membranes were blocked in 5% (*w*/*v*) non-fat milk for 1 h. Then, the membranes were probed with a primary antibody at 4 °C overnight, and incubated with HRP-conjugated secondary antibody. Finally, proteins were visualized using a BeyoECL Plus enhanced chemiluminescence system (Beyotime, Nanjing, China). The protein level was normalized to β-actin. Protein bands were quantified with Image J (Image 2×, NIH, Bethesda, MD, USA).

### 4.11. Statistical Analysis

Statistical analysis was performed using GraphPad Prism 5.0 (San Diego, CA, USA). The data were presented as mean ± SD. Statistical comparisons were performed using one-way analysis of variance. Value of *p* < 0.05 was considered statistically significant.

## 5. Conclusions

**Figure 8:**
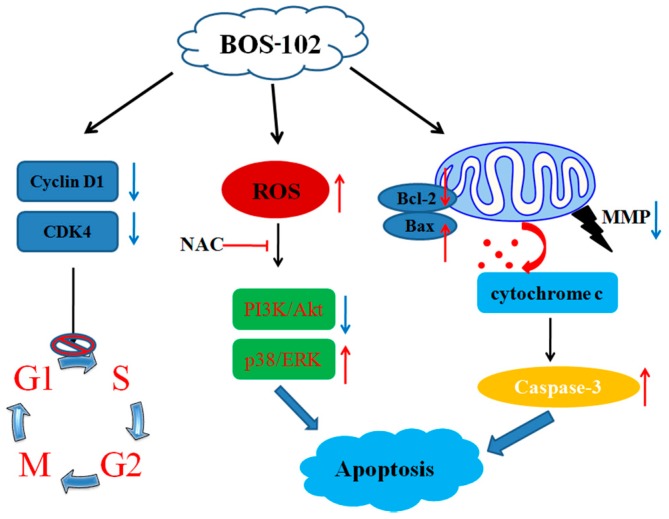
The proposed molecular mechanisms of cell cycle arrest and apoptosis induced by **BOS**-**102** in A549 cells.

In summary, our study extensively evaluated the anti-proliferative effect of **BOS**-**102** in human lung cancer cells, which was mediated through the arresting cell cycle in the G0/G1 phase and inducing cell apoptosis. Furthermore, our results provided evidence that **BOS**-**102** induced apoptosis in A549 cells through ROS-mediated inhibition of PI3K/Akt and activation of p38/ERK signaling pathways (Figure 8). Overall, our study indicated that the novel bromophenol derivative **BOS**-**102** has the potential to develop into an anticancer drug.
